# Difference in *TRI13* Gene Sequences between the 3-Acetyldeoxynivalenol Producing *Fusarium graminearum* Chemotypes from Canada and China

**DOI:** 10.3390/ijms12096164

**Published:** 2011-09-20

**Authors:** Chami Amarasinghe, Jian-Hua Wang, Yu-Cai Liao, W.G. Dilantha Fernando

**Affiliations:** 1 Department of Plant Science, University of Manitoba, Winnipeg, MB R3T2N2, Canada; E-Mail: umamarac@cc.umanitoba.ca; 2 Molecular Biotechnology Laboratory of Triticeae Crops, Huazhong Agricultural University, Wuhan 430070, Hubei, China; E-Mails: jianhuawang163@163.com (J.H.W.); yucailiao@mail.hzau.edu.cn (Y.-C.L.); 3 College of Plant Science and Technology, Huazhong Agricultural University, Wuhan 430070, Hubei, China

**Keywords:** *Fusarium graminearum*, chemotypes, PCR

## Abstract

Positive-negative PCR assays based on the genes involved in the trichothecene biosynthesis pathway are useful in assessing the risk of trichothecene contamination in grain and are important in epidemiological studies. A single PCR detection method based on the structural gene sequence of *TRI13* gene has been developed to predict the 3-ADON, 15-ADON and NIV chemotypes in China. The chemotypic differences are based on the deletions within the *TRI13* gene. The objective of this study was to assess the reliability of using this single primer based on the *TRI13* gene to differentiate the *F. graminearum* chemotypes in Canada. In this study, we found that, this single PCR detection method based on the deletions in the *TRI13* gene cannot be used to differentiate the 3-ADON and 15-ADON chemotypes in the Canadian *F. graminearum* isolates; further sequence analysis of the PCR products confirmed that both Canadian 3-ADON and 15-ADON chemotypes have the 61 bp deletion in the *TRI13* gene. This 61 bp deletion was absent in the Chinese 3-ADON isolates. Therefore these findings revealed that there are genetic differences between the examined 3-ADON *F. graminearum* isolates from Canada and China. The observed genetic differences between the 3-ADON chemotype populations in Canada and China may be resulted from a random mutation (insertion/deletion) that took place in one of the populations and accumulated due to genetic drift and/or selection.

## 1. Introduction

Fusarium head blight (FHB) is an economically important disease worldwide. Apart from causing significant yield losses the fungus can produce trichothecene mycotoxins. Harvested grains, contaminated with trichothecene mycotoxins can cause both acute and chronic side effects in livestock and humans. It has been reported that 25% of the world crop is affected by mycotoxins [1]. In plants, these mycotoxins have been shown to act as virulence factors during pathogenesis [2]. There are mainly two classes of trichothecenes; class A and class B, class B contains a keto group at the C-8 position of the trichothecene ring [3]. Different *Fusarium* spp such as, *F. graminearum*, *F. culmorum* and *F. cerealis* can produce different types of class B trichothecenes, such as deoxynivalenol (DON), nivalenol (NIV) and their acetylated derivatives, 3-acetyldeoxynivalenol (3-ADON), 15-acetyldeoxynivalenol (15-ADON) and 4-acetylnivalenol (4-ANIV). Based on type B tricothecene production, three different chemotypes of *F. graminearum* have been identified. The 3-ADON chemotype produces both DON and 3-ADON; 15-ADON chemotype produces both DON and 15-ADON; and the NIV chemotype produces NIV and 4-ANIV [3]. The differences between chemotypes are governed by the different genes involved in the trichothecene biosynthesis pathway. Trichothecene biosynthesis is a complex process, mediated by at least 10 genes that are involved in a series of oxygenation, isomerization and esterification steps [4]. The trichothecene gene cluster includes trichodiene synthase (*TRI5*), P450 oxygenase (*TRI4* and *TRI11*), acetyltransferases (*TRI3* and *TRI7*), transcription factors (*TRI6* and *TRI10*), a toxin efflux pump (*TRI12*), and two unidentified hypothetical proteins (*TRI8* and *TRI9*). The acetyltransferase gene *TRI101*, unlinked to the *TRI* cluster, is also found to be involved in the biosynthesis pathway. Two genes *TRI13* and *TRI14* have been identified from *F. sporotrichiodies* [4,5]. Among the *TRI* cluster genes *TRI13* is found to be the determinant for the DON-NIV switching in *Fusarium* and *TRI7* is involved in further modification of NIV to its acetylated derivative 4-ANIV [5]. NIV chemotypes have functional copies of *TRI13* and *TRI7* genes whereas both genes are non-functional in DON chemotypes [5].

Various PCR based assays have been developed to examine the trichothecene mycotoxin genotypes of *F. graminearum*. These assays are based on the amplification of a part of a gene that encodes for key enzymes involved in the trichiothecene biosynthesis pathway [6–9]. These PCR based assays provide a rapid and cost-effective way of identifying the different *F. graminearum* chemotypes [7].

The genotypic differences among the *F. graminearum* isolates collected from different populations in diverse geographic regions have been extensively studied [10]. To date, 13 phylogenetically distinct species have been identified within the *F. graminearum* clade based on DNA sequences from 13 genes [11–14]. These lineages are morphologically cryptic. *F. graminearum sensu stricto* (lineage 7) is the predominant species found in North America [15]. In China, *F. asiaticum* (lineage 6) and *F. graminearum sensu stricto* (lineage 7) are the most prevalent FHB causing species [16]. O’Donnell *et al.* [10] and Ward *et al.* [7] reported that the evolution of genes involved in the B-tricothecene biosynthesis does not correlate with the *F. graminearum* clade phylogeny. It is believed that chemotype polymorphism is trans-specific and has been maintained through multiple speciation events by balancing selection [7]. High levels of genetic variations have been found in the virulence associated genes within the *F. graminearum* clade [7]. The different lineages of *F. graminearum* have different geographic distributions; also differ in the type of mycotoxins that they produced and they may differ in their ability to cause disease on particular crops [17]. Environment factors within a certain geographical area could influence the prevalence of a particular chemotype. Zhang *et al.* [16] investigated the mycotoxin chemotype frequency of the *F. graminearum* strains isolated from wheat in FHB epidemic regions of China. The investigated chemotypes appeared to have different geographical distribution patterns and these were associated with the annual average temperatures in the region [17]. Qu *et al.* [18] also investigated the geographical distribution of *F. graminearum* and *F. asiaticum* isolates from all regions in China and demonstrated that *F. graminearum* isolates were more prevalent in the cooler regions whereas *F. asiaticum* in the warmer regions.

Ward *et al.* [7] reported the impact of environment on pathogen fitness. It has been reported that the chemotypes of *F. graminearum* from China and North America were different [3,19]. Miller *et al.* [3], analyzed the metabolic profiles of *F. graminearum* strains using gas chromatography/mass spectroscopy and reported that, chemotype 1A (DON and 3-ADON) was common in China and chemotype 1B (DON and 15-ADON) in North America. The results from phylogenetic studies are important to prevent the unintentional introduction of species or genetically different strains from one genetically unique population to another population especially with international trade [20].The introduction of new *Fusarium* species to the existing population may affect the current management strategies and breeding programs.

Therefore studies on the genetic diversity of *F. graminearum* chemotypes found in different geographical regions are important to prevent the future FHB epidemics. Wang *et al.* [21] developed a generic PCR detection method based on the *TRI13* gene to identify 3-ADON, 15-ADON and NIV chemotypes using a single primer pair. The objective of this study is to examine the applicability and reliability of this generic PCR detection based on *TRI13* gene to identify the chemotypes of *F. graminearum* isolates from Canada.

## 2. Materials and Methods

### 2.1. Fungal Isolation and DNA Extraction

*F. graminearum* isolates collected from different regions of Canada (120 isolates) and China (39 isolates) were used (Table 1). Some of the Canadian isolates were identified using multiplex PCR by Guo *et al.* [22] and the others by Dr.’s Ward and O’Donnell at the United States Department of Agriculture, Peoria, IL, using a multilocus genotyping assay. Chinese isolates were identified in Dr.’s Xu Zhang and Hongxiang Ma’s labs, at the Jiangsu Academy of Agriculture Science, China.

Single spore cultures of *F. graminearum* isolates growing from the *fusarium* damaged kernels were used for the DNA extraction. All isolates were grown on potato dextrose agar (PDA) plates for 7 days and genomic DNA was extracted from the freeze dried aerial mycelium using a CTAB based protocol described by Fernando *et al.* [23]. DNA was quantified using the NanoDrop3300 (ThermoFisher Scientific Inc.). DNA was diluted using sterilized distilled water for final concentration of 50 ng/μL.

### 2.2. SCAR Analysis

All the *Fusarium* strains were subjected to SCAR (sequence characterized amplified region) analysis with a pair of primers Fg16F/R [16,24]. These primers generate a 410 bp DNA fragment specific for SCAR group I and a 497 bp fragment for SCAR group V, respectively [16].

### 2.3. PCR Assays for Trichothecene Chemotypes

Two PCR assays were carried out. In the first PCR, the fungal DNA was amplified using the single primer set, developed by Wang *et al.* [21]. The single primer set used for amplification was as follows: TRI13P1 (5′-CTCSACCGCATCGAAGASTCTC-3′) and TRI13P2 (5′-GAASGTCGCARGACCTTGTTTC-3′), these primers generate a 859 bp fragment from NIV producing strains, a 644 bp fragment from 3-ADON producers and a 583 bp fragment from 15-ADON producers, respectively [21].

In the second PCR, the same DNA was amplified by the multiplex primer set developed by Ward *et al.* [25]. The primers for multiplex PCR were as follows: 3CON (5′-TGGCAAAGACTGGTTCAC-3′), 3D15A (5′-ACTGACCCAAGCTGCCATC-3′), 3D3A (5′-CGCATTGGCTAACACATG-3′) and 3NA (5′-GTGCACAGAATATACGAGC-3′) and they produce a 243 bp fragment for 3-ADON chemotypes, a 610 bp fragment for 15-ADON chemotypes and a 840 bp fragment for NIV chemotypes [25].

### 2.4. PCR Conditions

Both PCR assays were conducted using 50 ng of fungal DNA in a total volume of 25 μL containing 10 mM Tris HCl (pH 8.0), 1.25 mM MgCl_2_, 1 U Taq polymerase (Invitrogen, Carlsbad, CA, USA), 2.5 mM dNTPs and 10 mM from each primer. The PCR amplification of TRI13P1 and TRI13P2 primers consisted of an initial step at 94 °C for 4 min, followed by 35 cycles of 94 °C for 1 min, 58 °C for 40 s, 72 °C for 40 s, then a final extension at 72 °C for 6 min. The PCR amplification of multiplex primers consisted of an initial step at 94 °C for 5 min, followed by 45 cycles of 94 °C for 30 s, 52 °C for 30 s, 72 °C for 1 min, then a final extension of 72 °C for 8 min. Resulting PCR products were separated by 2% gel electrophoresis, stained with ethidium bromide (EtBr) at a final concentration of 0.2 μg/mL and visualized under UV light.

### 2.5. Confirmation of PCR Products Using Sequencing

A representative sample of the 120 Canadian isolates and 24 of the Chinese isolates were used for sequencing. Selected PCR products (4 Canadian 15-ADON isolates, 4 Canadian 3-ADON isolates, 5 Chinese 15-ADON isolates, 5 Chinese 3-ADON isolates and 4 Chinese NIV isolates,) were sequenced (Macrogene Corp, USA) and the sequences were subjected to multiple alignment using ClustalX (1.8) software to examine the expected deletions within the sequences.

## 3. Results

**Figure 1:**
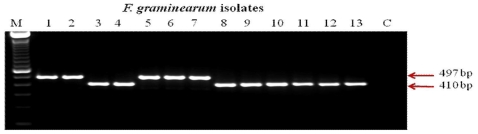
DNA of Chinese and Canadian *Fusarium graminearum* chemotypes amplified using Fg16 F/R primers. The 497 bp represents SCAR group V and 410 bp represents SCAR group I. Lane M: Marker; Lanes 1 and 2: NIV chemotypes, China (Fg-0921, 0905); Lanes 3 and 4: 15-ADON chemotype, China (Fg-1960, 0819); Lanes 5-7: 3-ADON chemotypes, China (Fg-0919, 0926, 0970); Lanes 8-10: 3-ADON chemotypes, Canada (M5-06-01, ON-06-39, DF-Fg-2); Lanes 11-13: 15-ADON chemotypes, Canada (DF-Fg- 144, ON-06-05, 55-1); Lane C: Control.

SCAR assays of all the strains from Canada and China with primer pair Fg16F/R revealed the presence of two SCAR groups, I and V. All the 3-ADON and 15-ADON chemotypes from Canada belong to the SCAR group I. All the NIV and 3-ADON chemotypes from China belong to SCAR group V. However, 15-ADON-producing isolates from China belong to either SCAR group I or V (Figure 1).

**Figure 2:**
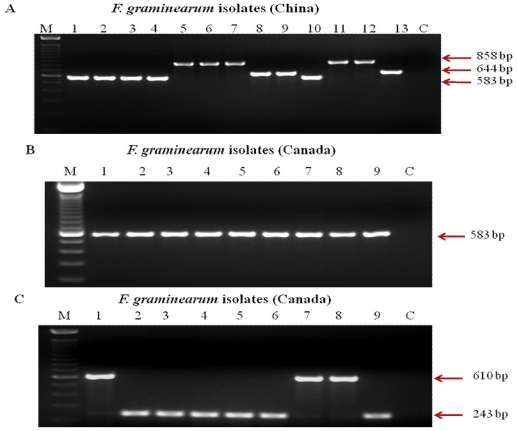
(**A**) DNA of Chinese *Fusarium graminearum* chemotype isolates amplified using TRI13P1/Tri13P2 primers [21]. The 583 bp fragments represented 15-ADON chemotypes, 644 bp represented 3-ADON chemotypes and 858 bp fragments represented NIV chemotypes. Lanes 1-4 and 10: 15-ADON chemotypes (Fg-0819, 1960, 0963, 0938, 0952); Lanes 8-9 and 13: 3-ADON chemotypes (Fg-0919, 0926, 0970); Lanes 5-7, 11 and 12: NIV chemotypes (Fg-0921, 0905, 0973, 0970, 0903); (**B**) DNA of Canadian chemotype isolates amplified using TRI13P1/Tri13P2 primers. The 583 bp fragments represented both 3-ADON and 15-ADON chemotypes. Lanes 1, 7 and 8: 15-ADON chemotypes (PEI-06- 34, ON-06-05, ON-06-17); Lanes 2-6 and 9: 3-ADON chemotypes (M8-06-05, M5-06-01, SIA-06-03, PEI-06-33, ON-06-39, DF-Fg-2); (**C**) DNA of Canadian chemotype isolates amplified using multiplex. The 610 bp fragments represented 15-ADON chemotypes and 243 bp fragments represented 3-ADON chemotype. Lanes 1, 7 and 8: 15-ADON chemotypes (PEI-06-34, ON-06-05, ON-06-17); Lanes 2-6 and 9: 3-ADON chemotypes (M8-06-05, M5-06-01, SIA-06-03, PEI-06-33, ON-06-39, DF-Fg-2).

**Figure 3:**
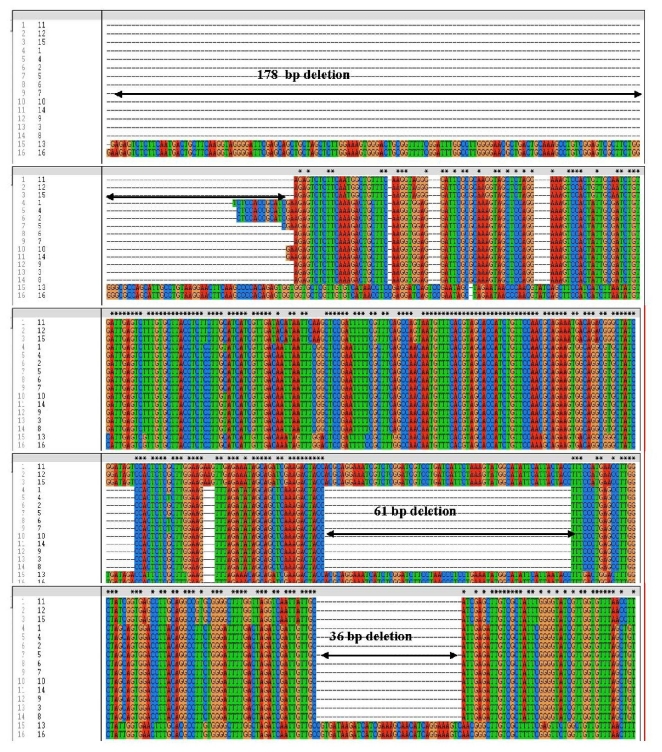
Alignments of *TRI13* gene sequences amplified from Canadian *Fusarium graminearum* 3-ADON, 15-ADON chemotypes and Chinese 3-ADON, 15-ADON and NIV chemotypes. 3-ADON chemotypes, Canada (1-M8-06-05, 2-ON-06-39, 3-Q-06-32, 7- 55-3), 15-ADON chemotypes, Canada (4-ON-06-17, 5-DF-Fg-144, 6-Q-06-10, 8-55-1), 3- ADON chemotypes, China (11-Fg-0919, 12-Fg-0926, 15-Fg-0970), 15-ADON chemotypes, China (9-Fg-0819, 10-Fg-1960, 14-Fg-0963), NIV chemotypes, China (13- Fg-0921, 16-Fg-0905).

One hundred and twenty *F. graminearum* isolates from different provinces in Canada, 24 *F. graminearum* isolates from China were selected to examine the reliability of the TRI13P1 and TRI13P2 primers for the identification of 3-ADON and 15-ADON chemotypes in Canadian *F. graminearum* isolates. Our PCR assay with TRI13P1/P2 primers revealed that this single primer pair cannot be used to differentiate the two chemotypes within the Canadian isolates, although they could identify the 3-ADON, 15-ADON and NIV isolates within Chinese isolates (Figure 2A). The TRI13P1 and TRI13P2 amplified a 583 bp fragment for all analyzed Canadian isolates regardless of whether they are 3-ADON or 15-ADON producers (Figure 2B). In this study all the above isolates were tested using multiplex PCR assay as well and it could differentiate the chemotypes within both Canadian and Chinese isolates. The multiplex primers amplified a 610 bp fragment for 15-ADON chemotypes, a 243 bp fragment for 3-ADON chemotypes (Figure 2C). The sequence analysis of the amplified fragments of the TRI13P1 and TRI13P2 primers of the selected 3-ADON, 15-ADON and NIV isolates from Canada, and China clearly showed the difference in *TRI13* gene (Figure 3). Compared with the NIV-producers, the 3-ADON chemotypes from Canada had both the 61 bp deletion and the 36 bp deletion whereas the 3-ADON chemotypes from China had only the 36 bp deletion; the 15-ADON chemotypes from both Canada and China had 36 bp and 61 bp deletions (Figure 3).

## 4. Discussion

Determination of the *F. graminearum* chemotypes using multiple primers makes the evaluation process tedious, time consuming and also they may generate false negative results [21,26]. Therefore use of a single primer that could differentiate the two chemotypes would accelerate the identification of chemotypes.

**Figure 4:**
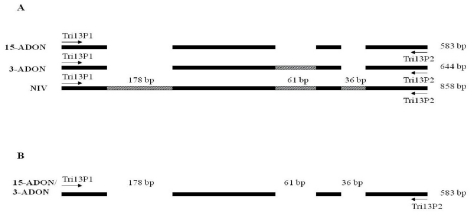
(**A**) Diagrammatic presentations of *TRI13* genes of 15-ADON, 3-ADON and NIV chemotypes in Chinese isolates of *Fusarium graminearum* (adopted from Wang *et al.* [21]); (**B**) Diagrammatic presentations of *TRI13* genes of 15-ADON and 3-ADON chemotypes in Canadian isolates of *F. graminearum*: ▭ - represent deletions; 
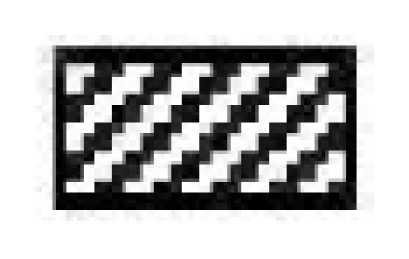
 - represent insertions in the *TRI13* gene.

The *TRI13* gene is functional only in NIV producers and encodes 3-acetyltrichothecene C-4 hydroxylase, enzyme that catalyses the C-4 oxygenation of calonectrin, disruption of the gene resulted in the loss of NIV production and accumulation of DON [5,27]. The *TRI13* gene consists of a unique intron of 62 bp. The TRI13P1/P2 primers were designed to identify the three chemotypes based on the deletions within the *TRI13* gene (Figure 4). The chemotypic identification is based on the three deletions within the *TRI13* gene found among the different chemotypes. The primers derived from *TRI13* gene has been used to identify the NIV producers and DON producers. This identification was based on the largest deletion of 178 bp fragment present only in DON producers but not in NIV producers [5,9,28,29]. The molecular differentiation between 3-ADON and 15-ADON chemotypes was based on the remaining two smaller deletions, 61 bp and 36 bp within the coding region. The 15-ADON chemotypes have both 61 bp and 36 bp deletions and amplified a product of 583 bp with TRI13P1/TRI13P2 primers; 3-ADON chemotypes have only the 36 bp deletion and amplified a product of 644 bp. In this study, all the examined Canadian isolates (both 3-ADON and 15-ADON) had amplified a product of 583 bp, revealing that Canadian 3-ADON chemotypes have the 61 bp deletion in the *TRI13* gene which is not present in the Chinese 3-ADON chemotypes. Sequencing results clearly confirmed the 61 bp deletion in the *TRI13* gene within the Canadian 3-ADON isolates. Lee *et al.* [5] reported that several deletions, substitutions and insertions were found in putative *TRI13* genes in DON producing strains collected from Korea, Nepal and the United states. Further, the nuclear alignment of the putative *TRI13* fragments indicated that these characteristics were highly conserved among the DON-producing strains from different geographical regions.

The multiplex PCR assay was based on the primers derived from trichothecene 15-O-acetyltransferase (*TRI3*) gene [7]. McCormick *et al.* [30] isolated *TRI3* gene from *F. sporotrichioides*. The *tri3* mutants of *F. sporotrichiodies* were able to acetylate the trichothecene C-3 hydroxyl group but not the C-15 hydroxyl group. These findings indicated that, acetylation of C-15 hydroxyl is mediated by *TRI3* gene. Multiplex PCR assay based on *TRI3* gene could differentiate the 3-ADON and 15-ADON chemotypes in the Canadian isolates that could not be differentiated using the TRI13P1/TRI13P2 primers (Figure 1A). So this confirmed that the *TRI13* gene sequences of the examined Canadian 3-ADON isolates are different from that of examined Chinese 3-ADON isolates. According to the SCAR analysis the Canadian 3-ADON isolates belong to SCAR group I and Chinese 3-ADON isolates SCAR group V. The isolates from SCAR group I represent *F. graminearum sensu stricto* lineage 7 whereas group V represents lineage 6 [16,29]. Therefore the single primer developed by Wang *et al.*, 2008 [21] based on the *TRI13* gene cannot be used to differentiate the 3-ADON and 15-ADON chemotypes of *F. graminearum sensu stricto* lineage 7 although it could be used to differentiate the three chemotypes of *F. graminearum* in lineage 6.

It has been reported that variations in trichothecene production may be caused by the alleleic polymorphisms in the tricothecene biosynthesis gene cluster, as a result of selection pressure from environments [7,10]. Several authors have examined the relationship between chemotype diversity and the geographical distribution. Both DON and NIV chemotypes have been reported in Africa, Asia and Europe whereas in North America only DON chemotypes were reported [3,8,31]. But in a recent study Starkey *et al.* [12] identified six *F. gramineaum* isolates with a NIV or 3-ADON chemotype in Louisiana, USA. Also Gale *et al.* [32] reported that NIV type populations of *F. graminearum* and *F. asiaticum* were prevalent on wheat in Southern Louisiana. They reported a presence of high proportion of NIV type *F. graminearum* among isolates (79%) collected from small grain growing regions of Louisiana and also NIV type isolates were identified in collections from Arkansas, North Carolina and Missouri. Studies done on phylogenetics of *Fusarium* spp. showed that significant levels of sexual recombination took place within the populations of the *F. graminearum* clade [10,33]. Members of all lineages are cross fertile with strains belonging to the lineage 7 and with strains in other lineages. Lineage 7 is considered as a universally cross-fertile lineage [34].The identification of a hybrid strain between two *F. graminearum* clade species [10] and reports by Bowden and Leslie [33] on the laboratory out-crossing amongst the lineages provided evidences for the possibility of developing novel lineages in an appropriate geographical location. The observed genetic differences between the 3-ADON chemotype populations in Canada and China may be resulted from a random mutation (insertion/deletion) that took place in one of the populations and accumulated due to genetic drift and/or selection. Climatic conditions, crop rotations and crop management strategies also differ markedly between Canada and China; therefore these also have an influence on the spread of the populations within a particular geographical area.

The genetic diversity of the species within the *F. graminearum* clade from different geographical regions in the world may affect the current disease management, quarantine regulations and breeding strategies [11]. Therefore studies on genetic diversity of *F. graminearum* clade species are important in preventing future outbreaks of FHB epidemics. The findings from this study provide a foundation for further investigations to understand the genetic diversity of *F. graminearum* chemotypes from different geographical regions.

## Figures and Tables

**Table 1:** Description of location, host and number of *Fusarium graminearum* isolates used in this study.

Location	Host	Number of Isolates
Manitoba, Canada	Wheat	93
Saskatchewan, Canada	Wheat	15
Nova Scotia, Canada	Wheat	3
Alberta, Canada	Wheat	4
Prince Edward Island, Canada	Wheat	2
Quebec, Canada	Wheat	3
China	Wheat	39
